# Hyperchloremia is associated with poor renal outcome after coronary artery bypass grafting

**DOI:** 10.1186/s12882-021-02554-0

**Published:** 2021-10-18

**Authors:** Jae Shin Choi, Donghwan Yun, Dong Ki Kim, Kook-Hwan Oh, Kwon Wook Joo, Yon Su Kim, Ki Young Na, Seung Seok Han

**Affiliations:** 1 Department of Internal Medicine, Pyeongtaek St. Mary’s Hospital, Gyeonggi-do, Korea; 2 Department of Biomedical Sciences, Seoul National University College of Medicine, Seoul, Korea; 3 Department of Internal Medicine, Seoul National University College of Medicine, 103 Daehakro, Jongno-gu, Seoul, 03080 Korea; 4 Department of Internal Medicine, Seoul National University Bundang Hospital, Gyeonggi-do, Korea

**Keywords:** Acute kidney injury, Coronary artery bypass grafting, End-stage renal disease, Hyperchloremia

## Abstract

**Background:**

Hyperchloremia is associated with the risks of several morbidities and mortality. However, its relationship with acute kidney injury (AKI) and end-stage renal disease (ESRD) in patients undergoing coronary artery bypass grafting (CABG) remains unresolved.

**Methods:**

A total of 2977 patients undergoing CABG between 2003 and 2015 were retrospectively reviewed from two tertiary hospitals. Patients were categorized by serum chloride levels into normochloremia (95–105 mmol/L), mild hyperchloremia (106–110 mmol/L), and severe hyperchloremia (> 110 mmol/L). The odds ratios (ORs) for AKI and hazard ratios (HRs) for ESRD were calculated after adjustment for multiple covariates. The death-adjusted risk of ESRD was additionally evaluated.

**Results:**

Postoperative AKI occurred in 798 patients (26.5%). The hyperchloremia group had a higher risk of AKI than the normochloremia group, wherein the risk was incremental depending on the severity of hyperchloremia, as follows: ORs were 1.26 (1.06–1.51) and 1.95 (1.52–2.51) in the mild and severe hyperchloremia groups, respectively. During a median period of 7 years (maximum 15 years), 70 patients (2.3%) had ESRD. The severe hyperchloremia group was at an elevated risk of ESRD compared with the normochloremia group, with an HR of 2.43 (1.28–4.63). Even after adjusting for the competing risk of death, hyperchloremia was associated with the risk of ESRD.

**Conclusions:**

Preoperative hyperchloremia is associated with poor renal outcomes such as AKI and ESRD after CABG. Accordingly, serum chloride should be monitored in patients undergoing CABG.

## Introduction

Electrolyte abnormalities are common in patients with various medical conditions and are associated with increased morbidities and mortality [1, 2]. Both acute and chronic abnormalities of electrolytes are directly associated with adverse events in the general population [3, 4], which have been primarily studied for sodium and potassium. Recent studies have found that an abnormal range of serum chloride is associated with several morbidities and mortality in some pathologic conditions. Hyperchloremia was associated with in-hospital mortality and acute kidney injury (AKI) in critically ill septic patients [5–7], patients with ischemic stroke and intracerebral hemorrhage [8, 9].

Coronary artery bypass grafting (CABG) is one of the most common surgical procedures, and it has been proven to improve the survival of patients with severe angina. The recent mortality rate is approximately 5% during the perioperative period, but this rate is highly dependent on comorbid diseases and postoperative complications. As a postoperative complication, AKI develops in up to 30% of cases after CABG [10], and it is a major cause of several morbidities and mortality. Despite a decreasing trend in its incidence, the current rate of AKI remains high [11], which finally leads to chronic kidney disease or end-stage renal disease (ESRD) [12]. Accordingly, the prediction of AKI is a critical issue in patients undergoing CABG. Electrolyte abnormalities frequently occur in patients undergoing CABG [13], but no studies have identified the predictive capacity of hyperchloremia on renal outcomes such as AKI and ESRD. Herein, we addressed this issue after adjustment for multiple covariates and the competing risk of death.

## Materials and methods

### Data source and study samples

Data on patients were retrospectively reviewed from two tertiary hospitals, namely, Seoul National University Hospital and Seoul National University Bundang Hospital. A total of 3089 patients consecutively underwent CABG between January 2003 and December 2015. We excluded patients who were less than 18 years old (*n* = 1), had baseline ESRD (*n* = 62), underwent redo-CABG (*n* = 8), and had hypochloremia (i.e., < 95 mmol/L) (*n* = 21). Consequently, 2997 patients were analyzed in the present study. Both on-pump CABG with cardiopulmonary bypass (CPB) and off-pump CABG were analyzed.

### Study variables

Demographic data including age, sex, body mass index, comorbidities such as hypertension, diabetes mellitus, myocardial infarction, stroke, and peripheral vascular disease, current smoking, and alcohol were obtained from the electronic medical records. Surgical information, such as the number of grafting arteries, CPB, and intra-aortic balloon pump, was obtained. The medications, such as angiotensin-converting enzyme inhibitors, angiotensin II receptor blockers, beta-blockers, diuretics, and statins were reviewed. The following laboratory findings were considered:white blood cells, hemoglobin, albumin, cholesterol, glucose, sodium, potassium, carbon dioxide, and chloride. The estimated glomerular filtration rate (eGFR) was calculated using the Chronic Kidney Disease Epidemiology Collaboration eq. [14]. Left ventricular ejection fraction was measured before CABG, using the biplane approach and modified Simpson method from apical imaging planes in the lateral decubitus position [15].

AKI was defined as an increase in serum creatinine by ≥0.3 mg/dl or ≥ 1.5 times above baseline creatinine at hospital admission, in adherence to the Kidney Disease Improving Global Outcomes guideline [16]. ESRD was defined as initiation of renal replacement therapy or kidney transplantation due to failure ofkidney function. This information was obtained from the Korean Renal Registry. All-cause death data were obtained from the National Database of Statistics Korea. The patients were followed until April 2019, except for the death-censored cases.

### Statistical analysis

All statistical analyses were performed using SPSS version 25.0 (IBM, Armonk, NY, USA) and R version 3.5.1 (R Foundation, Vienna, Austria, http://cran.r-project.org). Data are presented as percentages for categorical parameters. Means (± standard deviations) or medians (interquartile ranges) were used for continuous parameters according to the normal distribution. Patients were categorized according to serum chloride levels, such as normochloremia (95–105 mmol/L), mild hyperchloremia (106–110 mmol/L), and severe hyperchloremia (> 110 mmol/L) [17–19]. Comparisons between non-normally distributed continuous variables were performed using the Mann-Whitney U test. Multiple comparisons among the study groups were performed by the Kruskal-Wallis test followed by a post hoc test that was adjusted with a less significant difference correction. Odds ratios of AKI risk were calculated in the hyperchloremia group compared with the normochloremia group. Survival curves were drawn using the Kaplan-Meier method. The Cox proportional hazard model was used to calculate the hazard ratio of the ESRD risk according to the serum chloride levels. The additive generalized model with penalized splines was used to analyze the nonlinear relationship between serum chloride level and outcome. The competing risk analysis was conducted using a class of κ-sample test and a proportional hazard model (*cmprisk* package). Differences were considered significant if the *P* value was less than 0.05.

## Results

### Baseline characteristics

The mean age of the patients was 66 ± 10 years and 73.7% of patients were male. The mean value of eGFR was 70.1 ± 20.1 ml/min/1.73 m^2^. Forty-eight percent of patients had hyperchloremia at baseline, which was categorized into mild (37.3%) and severe (11.1%) hyperchloremia. Patients with hyperchloremia had a tendency to be older, female, diabetic, and anemic compared with normochloremic patients. The differences in other characteristics are shown in Table 1.

**Table 1:** Baseline characteristics of the study patients

Variables	Total (*n* = 2977)	NormoCl (*n* = 1524)	Mild HyperCl (*n* = 1118)	Severe HyperCl (*n* = 335)	*P*
Age (year)	65.5 ± 9.8	65.3 ± 10.0	65.5 ± 9.7	66.7 ± 9.6*	0.045
Male (%)	73.7	76.6	71.2^†^	68.4^†^	< 0.001
Body mass index (kg/m^2^)	24.3 ± 3.1	24.2 ± 3.2	24.5 ± 3.0^†^	24.4 ± 2.6	0.031
Systolic blood pressure (mmHg)	126.4 ± 20.6	127.1 ± 19.6	126.5 ± 21.4	123.9 ± 22.0*	0.038
Diastolic blood pressure (mmHg)	73.3 ± 12.4	73.9 ± 12.0	73.3 ± 12.7	70.9 ± 12.9^‡^	< 0.001
Comorbidities (%)					
Hypertension	57.5	55.2	58.3	65.4^†^	0.003
Diabetes mellitus	43.8	42.8	43.3	49.6*	0.075
History of myocardial infarction	9.3	8.2	10.1	11.6*	0.074
History of stroke	19.8	18.2	20.1	25.4^†^	0.011
History of peripheral vascular disease	6.5	5.4	7.0	9.9^†^	0.009
Smoking (%)	31.5	30.8	31.8	33.4	0.603
Alcohol (%)	29.7	30.0	29.3	29.3	0.923
Operating factor					
No. of grafting arteries	2.4 ± 0.8	2.5 ± 0.9	2.4 ± 0.7*	2.3 ± 0.7*	< 0.001
Cardiopulmonary bypass (%)	16.4	11.7	16.7^‡^	37.0^‡^	< 0.001
Intra-aortic balloon pump (%)	8.9	8.0	8.1	15.5^‡^	< 0.001
Surgical time (min)	345.5 ± 105.2	350.6 ± 113.0	345.3 ± 100.1	322.9 ± 79.5^‡^	< 0.001
Medications (%)					
ACE inhibitor or ARB	35.9	39.7	34.4^†^	23.9^‡^	< 0.001
Beta-blocker	38.3	39.2	38.7	33.1*	0.113
Diuretics	16.2	18.3	13.6^†^	15.5	0.005
Statin	40.5	43.4	39.8	30.1^‡^	< 0.001
Laboratory findings					
White blood cells (×10^3^/mm^3^)	7.8 ± 3.1	7.8 ± 3.0	7.8 ± 3.1	8.3 ± 3.1^†^	0.022
Hemoglobin (g/dL)	12.5 ± 2.1	13.1 ± 1.9	12.2 ± 2.0^‡^	10.9 ± 2.1^‡^	< 0.001
Albumin (g/dL)	3.8 ± 0.6	4.0 ± 0.6	3.7 ± 0.6^‡^	3.1 ± 0.7^‡^	< 0.001
Cholesterol (mg/dL)	149.4 ± 44.2	158.5 ± 41.3	147.0 ± 44.5^‡^	117.0 ± 39.8^‡^	< 0.001
Glucose (mg/dL)	140.5 ± 1.4	145.9 ± 1.9	128.9 ± 1.9	155.5 ± 8.5^‡^	< 0.001
eGFR (ml/min/1.73 m^2^)	70.1 ± 20.1	71.5 ± 19.8	70.3 ± 19.4	63.3 ± 21.7^‡^	< 0.001
Sodium (mmol/L)	139.6 ± 3.4	138.4 ± 2.9	140.6 ± 2.3^‡^	142.9 ± 3.1^‡^	< 0.001
Potassium (mmol/L)	4.2 ± 0.4	4.2 ± 0.4	4.1 ± 0.4^‡^	4.1 ± 0.5^‡^	< 0.001
Chloride (mmol/L)	105.5 ± 4.3	102.6 ± 2.3	107.5 ± 1.3^‡^	113.1 ± 2.7^‡^	< 0.001
Left ventricular EF < 40% (%)	12.6	14.3	11.3*	9.0^†^	0.007

NormoCl, normochloremia; HyperCl, hyperchloremia; ACE, angiotensin-converting enzyme; ARB, angiotensin II receptor blocker; eGFR, estimated glomerular filtration rate; EF, ejection fraction

*P <  0.05; ^†^*P* <  0.01; ^‡^*P* < 0.001 compared with the NormoCl group

### Risk of AKI in hyperchloremia

**Fig. 1:**
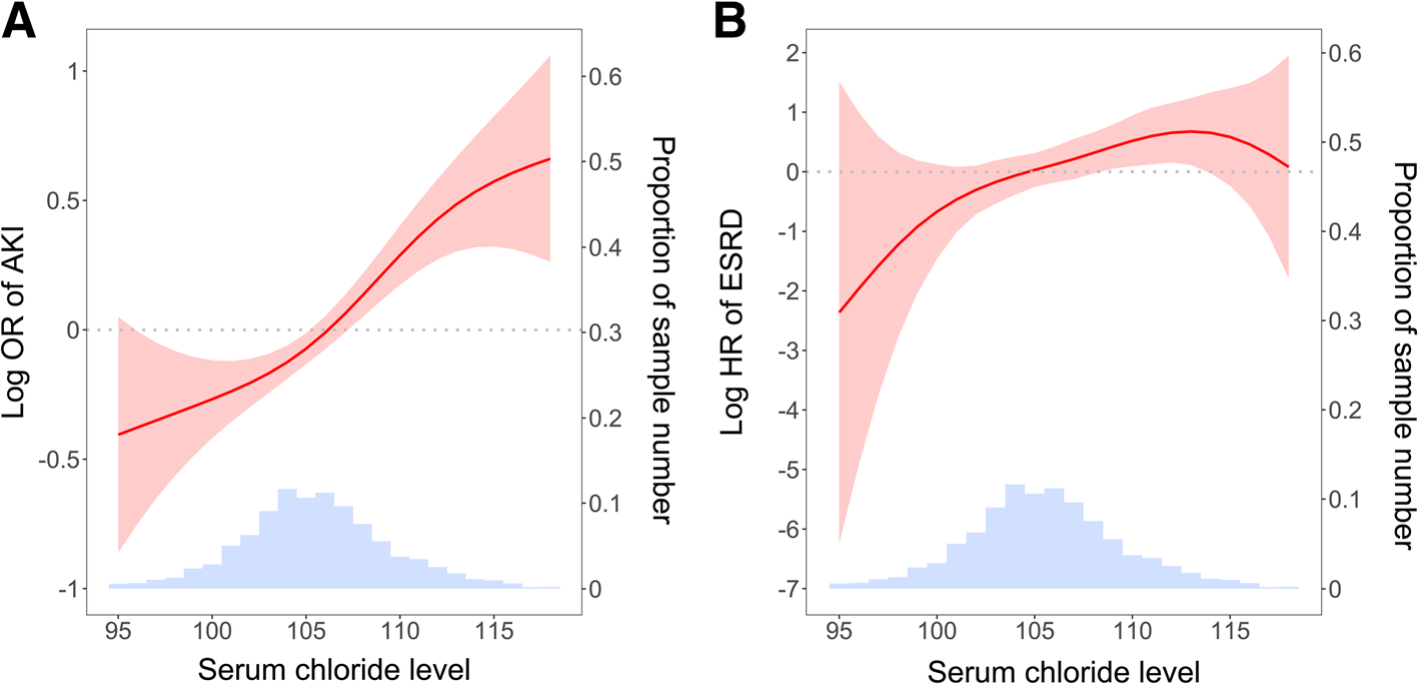
Nonlinear relationship between the predicted probability of renal outcomes and serum chloride levels. **A** Acute kidney injury (AKI). **B** End-stage renal disease (ESRD). The fitted line and 95% confidence intervals are indicated as red solid and shaded areas, respectively. Histogram of cases is indicated as the blue area. OR, odds ratio

Postoperative AKI occurred in 798 patients (26.5%). The hyperchloremic group had a higher risk of AKI than the normochloremic group (Table 2). This trend remained significant in despite of adjusting for multiple variables. When a nonlinear relationship was applied, the risk of AKI increased, depending on the increase in serum chloride (Fig. 1A). When the serum chloride level was considered a continuous variable in the multivariate regression model, the risk of AKI showed a 5% increase with each 1 mmol/L increase in the serum chloride level.

**Table 2:** Risk of acute kidney injury according to serum chloride levels

		Model 1		Model 2		Model 3	
Chloride groups	% of AKI	OR (95% CI)	*P*	OR (95% CI)	*P*	OR (95% CI)	*P*
NormoCl	23.2	1 (Reference)		1 (Reference)		1 (Reference)	
Mild HyperCl	27.5	1.26 (1.057–1.506)	0.010	1.19 (0.977–1.444)	0.084	1.20 (0.984–1.459)	0.073
Severe HyperCl	37.0	1.95 (1.516–2.507)	< 0.001	1.44 (1.064–1.961)	0.018	1.48 (1.086–2.017)	0.013

Model 1: Unadjusted

Model 2: Adjusted for variables with *P* < 0.05 in univariate analysis

Model 3: Adjusted for all the variables

AKI, acute kidney injury; OR, odds ratio; CI, confidence interval; NormoCl, normochloremia; HyperCl, hyperchloremia

### Risk of ESRD in hyperchloremia

**Fig. 2:**
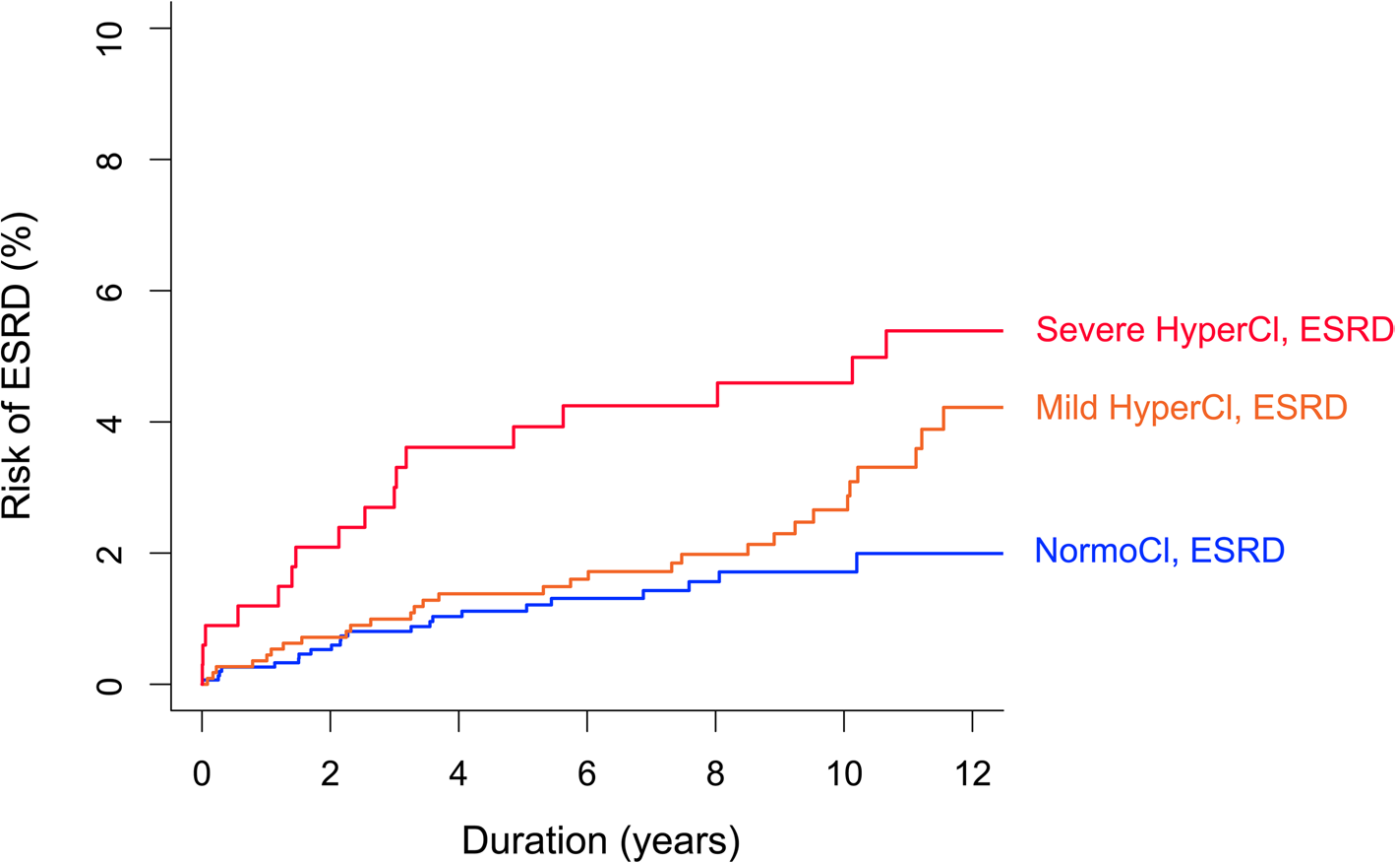
Kaplan-Meier curves of the risk of end-stage renal disease (ESRD) according to serum chloride levels. NormoCl, normochloremia; HyperCl, hyperchloremia

During the median period of 7 years (maximum 15 years), 70 patients (2.3%) had ESRD. Table 3 shows the HRs of ESRD according to the serum chloride levels. In both univariate and multivariate analyses, the severe hyperchloremic group had a higher risk of ESRD than the normochloremic group. When a nonlinear relationship was applied, the risk of ESRD increased depending on the increase in serum chloride (Fig. 1B). The Kaplan-Meier curves of ESRD support these results (Fig. 2). A 1 mmol/L increase in serum chloride conferred an 11.0% increase in the risk of ESRD.

**Table 3:** Risk of end-stage renal disease according to serum chloride levels

		Model 1		Model 2		Model 3	
Chloride groups	% of ESRD	HR (95% CI)	*P*	HR (95% CI)	*P*	HR (95% CI)	*P*
NormoCl	1.4	1 (Reference)		1 (Reference)		1 (Reference)	
Mild HyperCl	2.8	1.63 (0.943–2.824)	0.080	1.61 (0.907–2.867)	0.104	1.48 (0.821–2.658)	0.193
Severe HyperCl	5.1	2.43 (1.275–4.634)	0.007	1.43 (1.042–4.773)	0.039	2.34 (1.091–5.037)	0.029

Model 1: Unadjusted

Model 2: Adjusted for variables with *P* < 0.05 in univariate analysis

Model 3: Adjusted for all the variables

ESRD, end-stage renal disease; HR, hazard ratio; CI, confidence interval; NormoCl, normochloremia; HyperCl, hyperchloremia

During the follow-up period, 943 patients (31.7%) died. A competing risk analysis was performed because the association between hyperchloremia and ESRD could be affected by the high death rate [20]. The death rates were not different between the normochloremia and hyperchloremia groups (*P* = 0.094). Although the risk of death was adjusted, hyperchloremia remained a risk factor for ESRD. The adjusted HRs of ESRD in the mild and severe hyperchloremic groups were 1.70 (0.993–2.920) (*P* = 0.053) and 2.68 (1.401–5.140) (*P* = 0.003), respectively, compared with the normochloremic group. A 1 mmol/L increase in serum chloride conferred an 8.0% increase in the risk of ESRD (*P* = 0.003).

## Discussion

Chloride is an important anion in the extracellular fluid of humans. It accounts for approximately one-third of the tonicity and two-thirds of negatively charged ions in the plasma [21]. It plays a key role in the maintenance of acid-base balance and fluid homeostasis. The present study focused on hyperchloremia in patients undergoing CABG because it has been associated with worse outcomes in other medical conditions. Patients with hyperchloremia had higher risks of AKI and ESRD than those with normochloremia. This relationship was not affected by the death risk of patients. These results have clinical implications because serum chloride has merit in terms of easy and early measurement during the perioperative period.

Dyschloremia is identified in 25–45% of critically ill patients [7, 22]. Temporary hyperchloremia occurs more frequently than hypochloremia, specifically in up to 75% of critically ill patients during the first 24 h of hospital stay [23]. Hyperchloremia is primarily attributable to the loss of bicarbonates via gastrointestinal and renal paths. Additionally, it can be caused by dilution of bicarbonates through loading of fluids with a low bicarbonate concentration and excess infusion of chloride-rich fluids [24].

The physiological function of chloride includes regulation of extracellular and intracellular volume and acid-base homeostasis [24]. However, the pathophysiological role of abnormal chlorides is insufficiently understood because serum chloride levels have been less focused on in clinical or mechanistic studies. One study with 1940 septic patients found that hyperchloremia or its worsening trend was associated with high mortality [7]. Another study with 1221 critically ill patients revealed that hyperchloremia was prevalent during AKI compared to non-AKI [5]. This association was also identified in 1267 patients with acute intracerebral hemorrhage, wherein an increase in serum chloride, but not sodium, was associated with AKI [9]. Although electrolyte imbalance is frequent in patients undergoing CABG, no studies have confirmed the relationship between hyperchloremia and the risk of AKI and ESRD before the present study. This issue is important because both AKI and ESRD worsen the overall outcome and quality of life after CABG [12, 25, 26].

The association between hyperchloremia and kidney injury may be explained by the following hypotheses. Chlorides in the macular densa activate tubuloglomerular feedback and adjust renal efferent arterioles, but persistent hyperchloremia dysregulates tubuloglomerular feedback and vasoconstriction all of which reduce cortical perfusion [27]. Hyperchloremia results in chronic metabolic acidosis and then stimulates the production of angiotensin II and aldosterone to increase acid excretion, but chronic upregulation of these factors may cause tubulointerstitial inflammation and fibrosis [28]. Chloride may affect the function of the kidney and other organs because electrolytes play important roles in cellular metabolism and in the regulation of cellular membrane potentials, especially those of muscle and nerve cells in the heart.

The present result may warn the worse outcome when the patients receive therapy increasing serum chloride level before CABG. For example, we could prefer balanced crystalloids to 0.9% saline as an initial fluid because the latter has high chloride concentration. However, the present study design was observational, and thus, we could not strongly discuss this preventive issue. Future clinical trial will determine whether the chloride-lowering therapy is beneficial in hyperchloremic patients before CABG.

The strengths of our study are the large sample size and the long-term follow-up period, which enabled us to analyze the risk of ESRD. Nevertheless, the study has some limitations to be discussed. The study design was retrospective and observational in nature, which limited the understanding of causality between associations, although the aim of the study was to confirm the associations but not the causality. Follow-up chloride levels may be more important than the baseline levels, but the study did not collect them. Certain information related with AKI was not available, such as the aortic clamping time, and this could intervene in the associations.

## Conclusions

Preoperative hyperchloremia is associated with the risks of AKI and ESRD after undergoing CABG. Accordingly, monitoring serum chloride may be needed to predict renal outcomes more precisely. The study results provide a basis for future studies targeting chloride levels to predict and intervene in patients undergoing CABG.

## Data Availability

The datasets used and/or analyzed during the current study available from the corresponding author on reasonable request.
